# Crystal structure of di-μ-methano­lato-bis­{[*N*′-(1-benzoyl­prop-1-en-2-yl)thio­phene-2-carbohydrazidato-κ^3^ *O*,*N*′,*O*′]oxidovanadium(V)}

**DOI:** 10.1107/S1600536814020327

**Published:** 2014-09-27

**Authors:** Murilo C. Carroccia, Rafaela B. P. Pesci, Pedro Ivo da S. Maia, Victor M. Deflon

**Affiliations:** a Instituto de Química de São Carlos, Universidade de São Paulo, 13560-970, São Carlos, SP, Brazil; b Departamento de Química, Universidade Federal do Triângulo Mineiro, 38025-440, Uberaba, MG, Brazil

**Keywords:** crystal structure, thio­phene-2-carbohydrazide, vanadium(V) complex, dinuclear complex, alkoxide bridging

## Abstract

The neutral binuclear mol­ecule of the title complex, [V_2_(C_15_H_12_N_2_O_2_S)_2_(CH_3_O)_2_O_2_], exhibits inversion symmetry and consists of two oxidovanadium(V) (VO)^3+^ fragments, each coordinated by a dianionic and *O*,*N*′,*O*′-chelating *N*′-(1-benzoyl­prop-1-en-2-yl)thio­phene-2-carbohydrazidate ligand. The V^5+^ cations are bridged by two asymmetrically bonding methano­late ligands [V—O = 1.8155 (12) and 2.3950 (13) Å] originating from the deprotonation of the methanol solvent. The coordination sphere of the V^V^ atom is distorted octa­hedral, with the equatorial plane defined by the three donor atoms of the thio­phene-2-carbohydrazidate ligand and the O atom of a methano­late unit. The axial positions are occupied by the oxide group and the remaining methano­late ligand. The axially bound methano­late ligand shows a longer V—O bond length due to the *trans* influence caused by the tightly bonded oxide group. The packing of the complex mol­ecules is dominated by dispersion forces.

## Related literature   

For related structures of binuclear vanadium(V) complexes with *O*,*N*,*O*-chelating hydrazonate ligands and methano­late bridges, see: Sarkar & Pal (2009 ▶); Monfared *et al.* (2011 ▶); Maia *et al.* (2005 ▶, 2007 ▶). For synthetic details, see: Mondal *et al.* (2008 ▶).
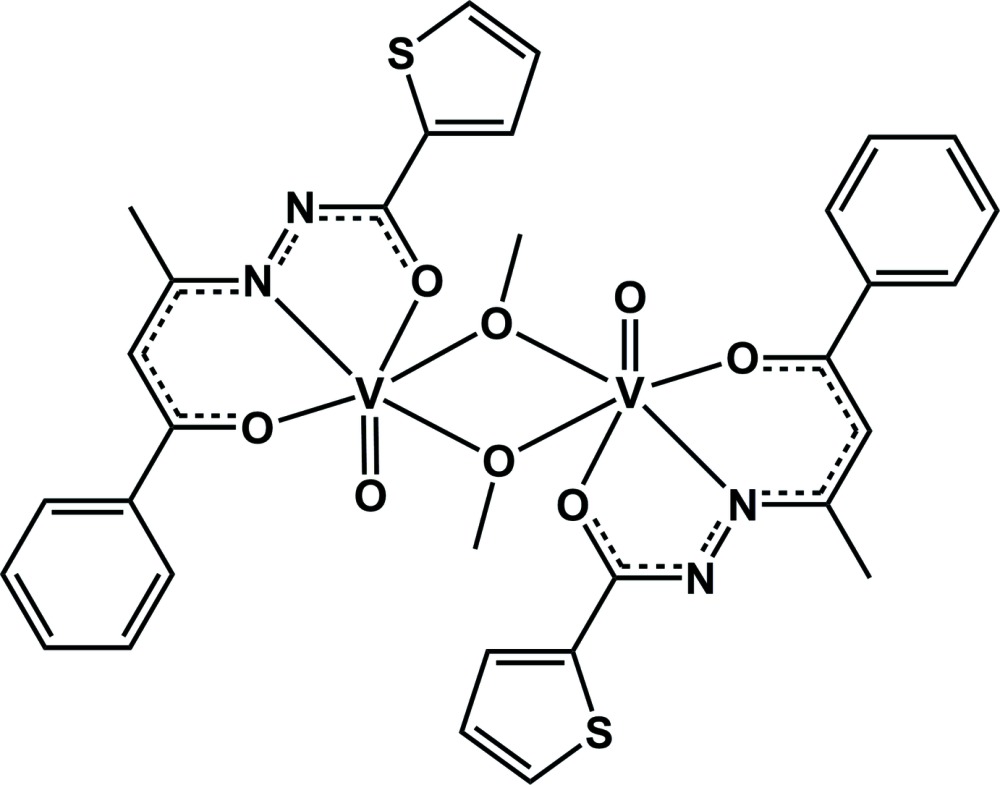


## Experimental   

### Crystal data  

[V_2_(C_15_H_12_N_2_O_2_S)_2_(CH_3_O)_2_O_2_]
*M* *_r_* = 764.60
Monoclinic, 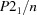

*a* = 10.9900 (2) Å
*b* = 15.9297 (3) Å
*c* = 11.0178 (3) Å
β = 119.884 (1)°
*V* = 1672.39 (6) Å^3^
*Z* = 2
Mo *K*α radiation
μ = 0.74 mm^−1^
*T* = 296 K
0.21 × 0.21 × 0.10 mm

### Data collection  

Bruker APEXII CCD diffractometer
Absorption correction: multi-scan (*SADABS*; Bruker, 2008 ▶) *T*_min_ = 0.860, *T*_max_ = 0.930
20108 measured reflections
3067 independent reflections
2718 reflections with *I* > 2σ(*I*)
*R*_int_ = 0.020

### Refinement  

*R*[*F* ^2^ > 2σ(*F* ^2^)] = 0.029
*wR*(*F* ^2^) = 0.083
*S* = 1.04
3067 reflections
219 parameters
H-atom parameters constrained
Δρ_max_ = 0.27 e Å^−3^
Δρ_min_ = −0.24 e Å^−3^

### 

Data collection: *APEX2* (Bruker, 2008 ▶); cell refinement: *SAINT* (Bruker, 2008 ▶); data reduction: *SAINT*; program(s) used to solve structure: *SHELXS97* (Sheldrick, 2008 ▶); program(s) used to refine structure: *SHELXL97* (Sheldrick, 2008 ▶); molecular graphics: *ORTEP-3 for Windows* (Farrugia, 2012 ▶) and *PLATON* (Spek, 2009 ▶); software used to prepare material for publication: *WinGX* (Farrugia, 2012 ▶).

## Supplementary Material

Crystal structure: contains datablock(s) global, I. DOI: 10.1107/S1600536814020327/wm5059sup1.cif

**Fig. 1.**
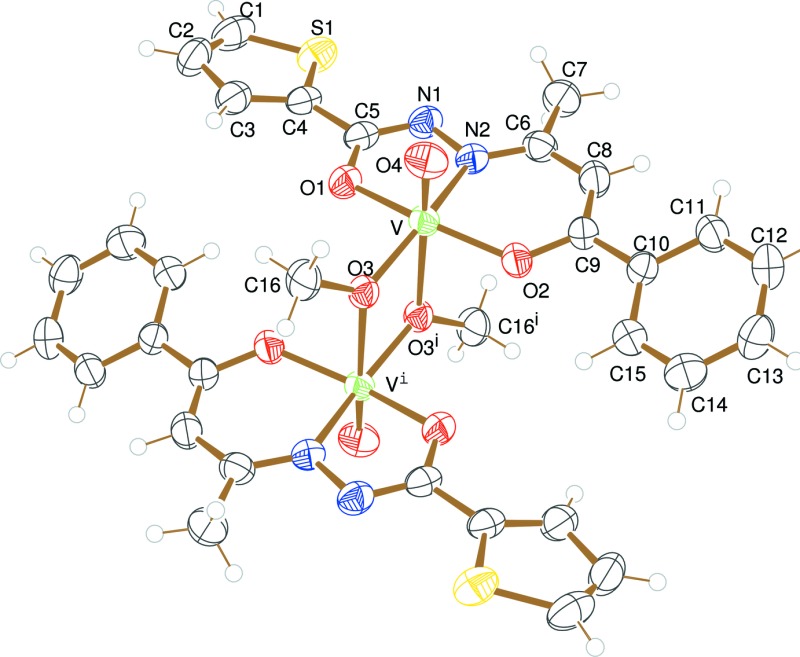
The binuclear molecular structure of the title compound with atom labels and displacement ellipsoids drawn at the 50% probability level. [Symmettry code: i) -x, -y+2, -z+1.]

Structure factors: contains datablock(s) I. DOI: 10.1107/S1600536814020327/wm5059Isup2.hkl

x y z . DOI: 10.1107/S1600536814020327/wm5059fig1.tif
The binuclear mol­ecular structure of the title compound with atom labels and displacement ellipsoids drawn at the 50% probability level. [Symmettry code: i) −*x*, −*y* + 2, −*z* + 1.]

**Fig. 2.**
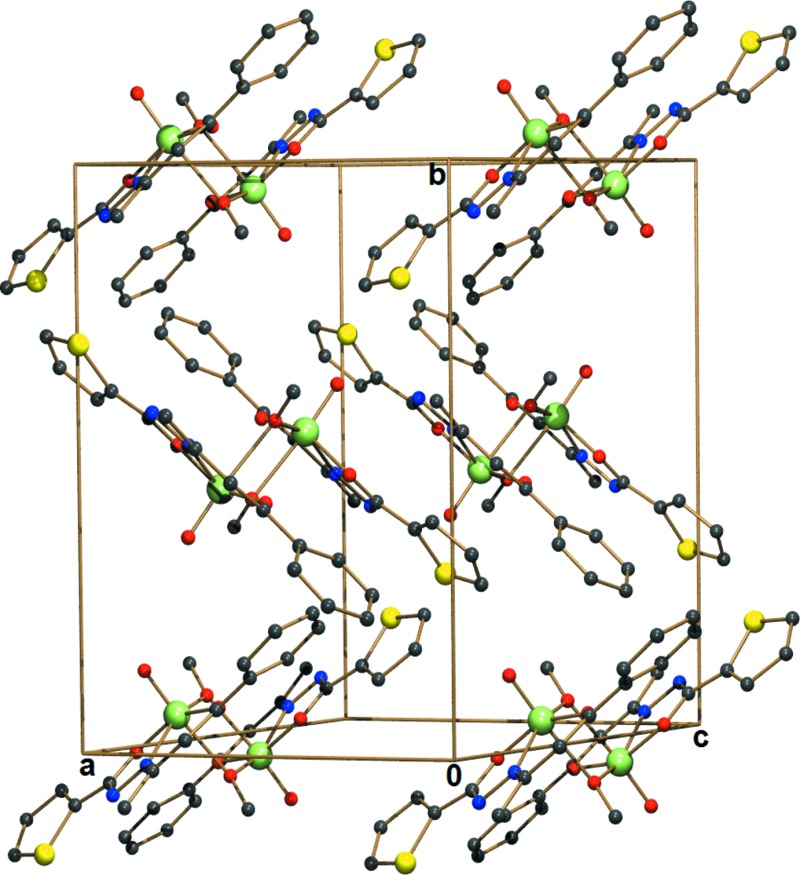
Packing diagram of the title complex. No hydrogen-bonding interactions are observed.

. DOI: 10.1107/S1600536814020327/wm5059fig2.tif
Packing diagram of the title complex. No hydrogen-bonding inter­actions are observed.

CCDC reference: 1023545

Additional supporting information:  crystallographic information; 3D view; checkCIF report

## supplementary crystallographic information

### S1. Experimental

The synthesis of the complex was developed by a slight modification of the procedure previously described by Mondal *et al.* (2008). 0.2 mmol (0.053 g) of [VO(acac)_2_] and 0.2 mmol of benzoylacetone-2-thinoylhydrazone (0.058 g) were diluted separately in methanol. The solutions were mixed and stirred for 0.5 h. A brown solution was obtained and after slow evaporation of the solvent single crystals were formed.

### S2. Refinement

The H atoms were positioned geometrically and refined using a riding model with C—H bond lengths of 0.96 Å (methyl) and of 0.93 Å (aromatic) and with *U*_iso_(H) = 1.5*U*_eq_(C) (methyl), and with *U*_iso_(H) = 1.2*U*_eq_(C) (aromatic).

### Crystal data

**Table:**

[V_2_(C_15_H_12_N_2_O_2_S)_2_(CH_3_O)_2_O_2_]	*F*(000) = 784
*M**_r_* = 764.60	*D*_x_ = 1.518 Mg m^−^^3^
Monoclinic, *P*2_1_/*n*	Melting point: 451 K
Hall symbol: -P 2yn	Mo *K*α radiation, λ = 0.71073 Å
*a* = 10.9900 (2) Å	Cell parameters from 9900 reflections
*b* = 15.9297 (3) Å	θ = 2.5–25.4°
*c* = 11.0178 (3) Å	µ = 0.74 mm^−^^1^
β = 119.884 (1)°	*T* = 296 K
*V* = 1672.39 (6) Å^3^	Prism, brown
*Z* = 2	0.21 × 0.21 × 0.10 mm

### Data collection

**Table:**

Bruker APEXII CCD diffractometer	3067 independent reflections
Radiation source: fine-focus sealed tube	2718 reflections with *I* > 2σ(*I*)
Graphite monochromator	*R*_int_ = 0.020
φ and ω scans	θ_max_ = 25.4°, θ_min_ = 2.1°
Absorption correction: multi-scan (*SADABS*; Bruker, 2008)	*h* = −13→13
*T*_min_ = 0.860, *T*_max_ = 0.930	*k* = −19→19
20108 measured reflections	*l* = −10→13

### Refinement

**Table:**

Refinement on *F*^2^	Primary atom site location: structure-invariant direct methods
Least-squares matrix: full	Secondary atom site location: difference Fourier map
*R*[*F*^2^ > 2σ(*F*^2^)] = 0.029	Hydrogen site location: inferred from neighbouring sites
*wR*(*F*^2^) = 0.083	H-atom parameters constrained
*S* = 1.04	*w* = 1/[σ^2^(*F*_o_^2^) + (0.0427*P*)^2^ + 0.7671*P*] where *P* = (*F*_o_^2^ + 2*F*_c_^2^)/3
3067 reflections	(Δ/σ)_max_ = 0.001
219 parameters	Δρ_max_ = 0.27 e Å^−^^3^
0 restraints	Δρ_min_ = −0.24 e Å^−^^3^

### Special details

**Table:**

Geometry. All e.s.d.'s (except the e.s.d. in the dihedral angle between two l.s. planes) are estimated using the full covariance matrix. The cell e.s.d.'s are taken into account individually in the estimation of e.s.d.'s in distances, angles and torsion angles; correlations between e.s.d.'s in cell parameters are only used when they are defined by crystal symmetry. An approximate (isotropic) treatment of cell e.s.d.'s is used for estimating e.s.d.'s involving l.s. planes.
Refinement. Refinement of *F*^2^ against all reflections. The weighted *R*-factor *wR* and goodness of fit *S* are based on *F*^2^, conventional *R*-factors *R* are based on *F*, with *F* set to zero for negative *F*^2^. The threshold expression of *F*^2^ > σ(*F*^2^) is used only for calculating *R*-factors(gt) *etc*. and is not relevant to the choice of reflections for refinement. *R*-factors based on *F*^2^ are statistically about twice as large as those based on *F*, and *R*- factors based on all data will be even larger.

### Fractional atomic coordinates and isotropic or equivalent isotropic displacement parameters (Å^2^)

**Table:**

	*x*	*y*	*z*	*U*_iso_*/*U*_eq_	
V	−0.01766 (3)	0.95609 (2)	0.63227 (3)	0.03545 (12)	
S1	−0.33170 (6)	1.19868 (4)	0.71561 (6)	0.05580 (17)	
O2	0.17270 (14)	0.93610 (9)	0.73088 (13)	0.0449 (3)	
O3	−0.06694 (12)	0.93265 (8)	0.45226 (12)	0.0362 (3)	
O4	−0.07281 (16)	0.87680 (9)	0.67733 (15)	0.0525 (4)	
N2	0.02549 (16)	1.03306 (10)	0.80379 (16)	0.0382 (4)	
N1	−0.08298 (17)	1.08675 (10)	0.78577 (17)	0.0426 (4)	
C9	0.26550 (19)	0.93078 (12)	0.86578 (19)	0.0387 (4)	
C8	0.2507 (2)	0.97723 (14)	0.9611 (2)	0.0463 (5)	
H7	0.3208	0.9727	1.0544	0.056*	
C6	0.1366 (2)	1.03221 (13)	0.9298 (2)	0.0417 (4)	
C7	0.1447 (2)	1.08777 (15)	1.0428 (2)	0.0539 (5)	
H2	0.2270	1.0740	1.1299	0.081*	
H3	0.0628	1.0797	1.0515	0.081*	
H1	0.1496	1.1453	1.0198	0.081*	
C5	−0.1873 (2)	1.07576 (12)	0.6604 (2)	0.0389 (4)	
O1	−0.18078 (14)	1.02693 (9)	0.56828 (14)	0.0436 (3)	
C4	−0.3193 (2)	1.11904 (12)	0.6175 (2)	0.0404 (4)	
C1	−0.5054 (3)	1.21073 (16)	0.6013 (3)	0.0607 (6)	
H6	−0.5611	1.2508	0.6118	0.073*	
C2	−0.5541 (2)	1.15642 (17)	0.4942 (3)	0.0603 (6)	
H4	−0.6476	1.1540	0.4238	0.072*	
C3	−0.4478 (2)	1.10288 (14)	0.4995 (2)	0.0473 (5)	
H5	−0.4625	1.0625	0.4326	0.057*	
C10	0.38325 (19)	0.87296 (12)	0.89970 (19)	0.0380 (4)	
C15	0.4093 (2)	0.84603 (14)	0.7951 (2)	0.0473 (5)	
H12	0.3543	0.8661	0.7043	0.057*	
C14	0.5155 (2)	0.79008 (16)	0.8240 (2)	0.0586 (6)	
H11	0.5325	0.7730	0.7532	0.070*	
C13	0.5968 (2)	0.75917 (15)	0.9579 (2)	0.0552 (6)	
H10	0.6685	0.7212	0.9773	0.066*	
C12	0.5717 (2)	0.78462 (14)	1.0622 (2)	0.0506 (5)	
H9	0.6258	0.7630	1.1520	0.061*	
C11	0.4678 (2)	0.84163 (13)	1.03576 (19)	0.0453 (5)	
H8	0.4534	0.8595	1.1079	0.054*	
C16	−0.1838 (2)	0.88198 (14)	0.3600 (2)	0.0493 (5)	
H15	−0.1841	0.8314	0.4072	0.074*	
H13	−0.1768	0.8680	0.2789	0.074*	
H14	−0.2692	0.9124	0.3317	0.074*	

### Atomic displacement parameters (Å^2^)

**Table:**

	*U*^11^	*U*^22^	*U*^33^	*U*^12^	*U*^13^	*U*^23^
V	0.03501 (19)	0.0397 (2)	0.03146 (18)	0.00311 (13)	0.01640 (14)	−0.00117 (12)
S1	0.0529 (3)	0.0550 (3)	0.0687 (4)	0.0045 (3)	0.0372 (3)	−0.0054 (3)
O2	0.0387 (7)	0.0601 (9)	0.0324 (7)	0.0123 (6)	0.0151 (6)	0.0024 (6)
O3	0.0319 (7)	0.0404 (7)	0.0327 (6)	−0.0024 (5)	0.0133 (5)	−0.0056 (5)
O4	0.0642 (10)	0.0465 (8)	0.0544 (8)	−0.0015 (7)	0.0355 (8)	0.0020 (7)
N2	0.0374 (9)	0.0420 (9)	0.0391 (8)	0.0022 (7)	0.0220 (7)	−0.0014 (7)
N1	0.0407 (9)	0.0434 (9)	0.0484 (9)	0.0061 (7)	0.0258 (8)	−0.0007 (7)
C9	0.0328 (10)	0.0459 (11)	0.0339 (9)	0.0006 (8)	0.0141 (8)	0.0041 (8)
C8	0.0390 (11)	0.0566 (12)	0.0359 (10)	0.0057 (9)	0.0131 (8)	−0.0022 (9)
C6	0.0444 (11)	0.0468 (11)	0.0368 (10)	−0.0043 (9)	0.0224 (9)	−0.0040 (8)
C7	0.0558 (13)	0.0607 (14)	0.0454 (11)	−0.0017 (11)	0.0256 (10)	−0.0133 (10)
C5	0.0395 (10)	0.0371 (10)	0.0486 (11)	0.0018 (8)	0.0285 (9)	0.0045 (8)
O1	0.0362 (7)	0.0522 (8)	0.0435 (7)	0.0058 (6)	0.0207 (6)	−0.0018 (6)
C4	0.0411 (11)	0.0397 (10)	0.0504 (11)	0.0018 (8)	0.0304 (9)	0.0054 (8)
C1	0.0559 (14)	0.0571 (14)	0.0868 (17)	0.0149 (11)	0.0489 (14)	0.0092 (13)
C2	0.0365 (11)	0.0722 (16)	0.0692 (15)	0.0081 (11)	0.0242 (11)	0.0161 (13)
C3	0.0426 (11)	0.0502 (12)	0.0530 (11)	0.0017 (9)	0.0268 (10)	0.0031 (10)
C10	0.0314 (9)	0.0420 (10)	0.0370 (9)	−0.0013 (8)	0.0144 (8)	0.0006 (8)
C15	0.0434 (11)	0.0582 (13)	0.0381 (10)	0.0049 (10)	0.0186 (9)	0.0035 (9)
C14	0.0568 (14)	0.0690 (15)	0.0573 (13)	0.0109 (12)	0.0341 (11)	−0.0018 (11)
C13	0.0409 (12)	0.0560 (13)	0.0616 (13)	0.0113 (10)	0.0202 (10)	0.0004 (11)
C12	0.0414 (11)	0.0503 (12)	0.0429 (11)	0.0047 (9)	0.0079 (9)	0.0019 (9)
C11	0.0433 (11)	0.0535 (12)	0.0335 (9)	0.0052 (9)	0.0150 (8)	0.0005 (8)
C16	0.0420 (11)	0.0522 (12)	0.0445 (11)	−0.0140 (9)	0.0145 (9)	−0.0093 (9)

### Geometric parameters (Å, º)

**Table:**

V—O4	1.5839 (15)	C5—O1	1.308 (2)
V—O3	1.8155 (12)	C5—C4	1.456 (3)
V—O2	1.8421 (13)	C4—C3	1.386 (3)
V—O1	1.9300 (13)	C1—C2	1.341 (4)
V—N2	2.0992 (16)	C1—H6	0.9300
V—O3^i^	2.3950 (13)	C2—C3	1.425 (3)
S1—C1	1.695 (3)	C2—H4	0.9300
S1—C4	1.715 (2)	C3—H5	0.9300
O2—C9	1.321 (2)	C10—C15	1.387 (3)
O3—C16	1.426 (2)	C10—C11	1.404 (3)
O3—V^i^	2.3950 (13)	C15—C14	1.373 (3)
N2—C6	1.315 (3)	C15—H12	0.9300
N2—N1	1.399 (2)	C14—C13	1.380 (3)
N1—C5	1.295 (3)	C14—H11	0.9300
C9—C8	1.359 (3)	C13—C12	1.370 (3)
C9—C10	1.477 (3)	C13—H10	0.9300
C8—C6	1.424 (3)	C12—C11	1.372 (3)
C8—H7	0.9300	C12—H9	0.9300
C6—C7	1.494 (3)	C11—H8	0.9300
C7—H2	0.9600	C16—H15	0.9600
C7—H3	0.9600	C16—H13	0.9600
C7—H1	0.9600	C16—H14	0.9600
			
O4—V—O3	103.06 (7)	N1—C5—C4	119.43 (17)
O4—V—O2	100.18 (7)	O1—C5—C4	117.45 (17)
O3—V—O2	103.99 (6)	C5—O1—V	117.66 (12)
O4—V—O1	98.62 (7)	C3—C4—C5	126.90 (19)
O3—V—O1	90.23 (6)	C3—C4—S1	111.43 (15)
O2—V—O1	153.09 (7)	C5—C4—S1	121.67 (15)
O4—V—N2	97.68 (7)	C2—C1—S1	112.87 (18)
O3—V—N2	156.10 (6)	C2—C1—H6	123.6
O2—V—N2	83.64 (6)	S1—C1—H6	123.6
O1—V—N2	74.89 (6)	C1—C2—C3	113.0 (2)
O4—V—O3^i^	174.44 (6)	C1—C2—H4	123.5
O3—V—O3^i^	71.92 (6)	C3—C2—H4	123.5
O2—V—O3^i^	79.07 (6)	C4—C3—C2	111.0 (2)
O1—V—O3^i^	83.98 (5)	C4—C3—H5	124.5
N2—V—O3^i^	87.73 (5)	C2—C3—H5	124.5
C1—S1—C4	91.68 (11)	C15—C10—C11	118.46 (18)
C9—O2—V	133.45 (13)	C15—C10—C9	120.02 (17)
C16—O3—V	124.60 (12)	C11—C10—C9	121.48 (17)
C16—O3—V^i^	121.85 (11)	C14—C15—C10	120.71 (19)
V—O3—V^i^	108.08 (6)	C14—C15—H12	119.6
C6—N2—N1	115.69 (16)	C10—C15—H12	119.6
C6—N2—V	128.24 (13)	C15—C14—C13	120.2 (2)
N1—N2—V	115.79 (12)	C15—C14—H11	119.9
C5—N1—N2	107.93 (15)	C13—C14—H11	119.9
O2—C9—C8	120.78 (18)	C12—C13—C14	119.8 (2)
O2—C9—C10	114.37 (16)	C12—C13—H10	120.1
C8—C9—C10	124.84 (17)	C14—C13—H10	120.1
C9—C8—C6	125.28 (18)	C13—C12—C11	120.78 (19)
C9—C8—H7	117.4	C13—C12—H9	119.6
C6—C8—H7	117.4	C11—C12—H9	119.6
N2—C6—C8	120.30 (17)	C12—C11—C10	120.02 (19)
N2—C6—C7	120.84 (19)	C12—C11—H8	120.0
C8—C6—C7	118.85 (18)	C10—C11—H8	120.0
C6—C7—H2	109.5	O3—C16—H15	109.5
C6—C7—H3	109.5	O3—C16—H13	109.5
H2—C7—H3	109.5	H15—C16—H13	109.5
C6—C7—H1	109.5	O3—C16—H14	109.5
H2—C7—H1	109.5	H15—C16—H14	109.5
H3—C7—H1	109.5	H13—C16—H14	109.5
N1—C5—O1	123.12 (17)		
			
O4—V—O2—C9	−63.04 (19)	C9—C8—C6—N2	9.1 (3)
O3—V—O2—C9	−169.38 (18)	C9—C8—C6—C7	−171.7 (2)
O1—V—O2—C9	70.6 (2)	N2—N1—C5—O1	−5.0 (2)
N2—V—O2—C9	33.66 (18)	N2—N1—C5—C4	174.88 (16)
O3^i^—V—O2—C9	122.57 (19)	N1—C5—O1—V	9.3 (2)
O4—V—O3—C16	28.39 (16)	C4—C5—O1—V	−170.54 (12)
O2—V—O3—C16	132.56 (15)	O4—V—O1—C5	88.96 (14)
O1—V—O3—C16	−70.51 (15)	O3—V—O1—C5	−167.79 (13)
N2—V—O3—C16	−121.15 (18)	O2—V—O1—C5	−45.0 (2)
O3^i^—V—O3—C16	−154.11 (17)	N2—V—O1—C5	−6.73 (13)
O4—V—O3—V^i^	−177.50 (7)	O3^i^—V—O1—C5	−96.00 (13)
O2—V—O3—V^i^	−73.33 (7)	N1—C5—C4—C3	−166.79 (19)
O1—V—O3—V^i^	83.59 (6)	O1—C5—C4—C3	13.1 (3)
N2—V—O3—V^i^	32.96 (16)	N1—C5—C4—S1	12.3 (3)
O3^i^—V—O3—V^i^	0.0	O1—C5—C4—S1	−167.85 (14)
O4—V—N2—C6	80.91 (18)	C1—S1—C4—C3	−0.08 (16)
O3—V—N2—C6	−128.97 (18)	C1—S1—C4—C5	−179.28 (17)
O2—V—N2—C6	−18.55 (17)	C4—S1—C1—C2	1.0 (2)
O1—V—N2—C6	177.82 (18)	S1—C1—C2—C3	−1.6 (3)
O3^i^—V—N2—C6	−97.81 (17)	C5—C4—C3—C2	178.37 (19)
O4—V—N2—N1	−92.68 (13)	S1—C4—C3—C2	−0.8 (2)
O3—V—N2—N1	57.4 (2)	C1—C2—C3—C4	1.5 (3)
O2—V—N2—N1	167.86 (13)	O2—C9—C10—C15	16.0 (3)
O1—V—N2—N1	4.23 (12)	C8—C9—C10—C15	−162.7 (2)
O3^i^—V—N2—N1	88.60 (12)	O2—C9—C10—C11	−161.71 (19)
C6—N2—N1—C5	−175.49 (17)	C8—C9—C10—C11	19.6 (3)
V—N2—N1—C5	−1.07 (19)	C11—C10—C15—C14	0.0 (3)
V—O2—C9—C8	−32.1 (3)	C9—C10—C15—C14	−177.8 (2)
V—O2—C9—C10	149.16 (14)	C10—C15—C14—C13	0.7 (4)
O2—C9—C8—C6	2.4 (3)	C15—C14—C13—C12	−0.2 (4)
C10—C9—C8—C6	−179.02 (19)	C14—C13—C12—C11	−1.0 (4)
N1—N2—C6—C8	177.40 (17)	C13—C12—C11—C10	1.7 (3)
V—N2—C6—C8	3.8 (3)	C15—C10—C11—C12	−1.1 (3)
N1—N2—C6—C7	−1.8 (3)	C9—C10—C11—C12	176.60 (19)
V—N2—C6—C7	−175.38 (15)		

Symmetry code: (i) −*x*, −*y*+2, −*z*+1.
