# Supplementary figures and images for: Crystal structure of di-μ-methano­lato-bis­{[N′-(1-benzoyl­prop-1-en-2-yl)thio­phene-2-carbohydrazidato-κ3 O,N′,O′]oxidovanadium(V)}
Source: Acta Crystallogr Sect E Struct Rep Online. 2014 Sep 27;70(Pt 10):m353–4. doi: 10.1107/S1600536814020327 (PMC4257161; doi:10.1107/S1600536814020327)

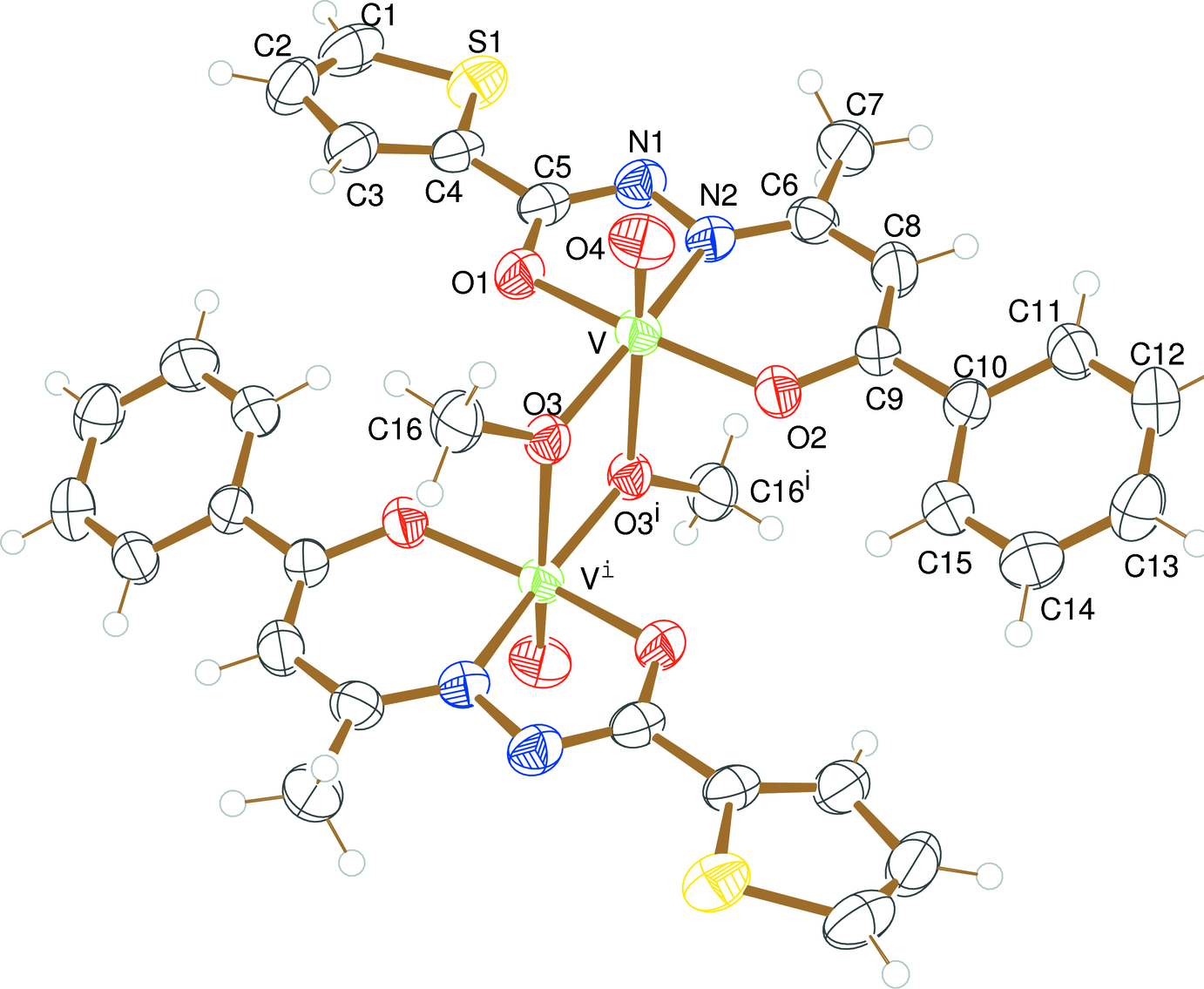

Supplement: Supplementary file 3 [file e-70-0m353-fig1.tif]

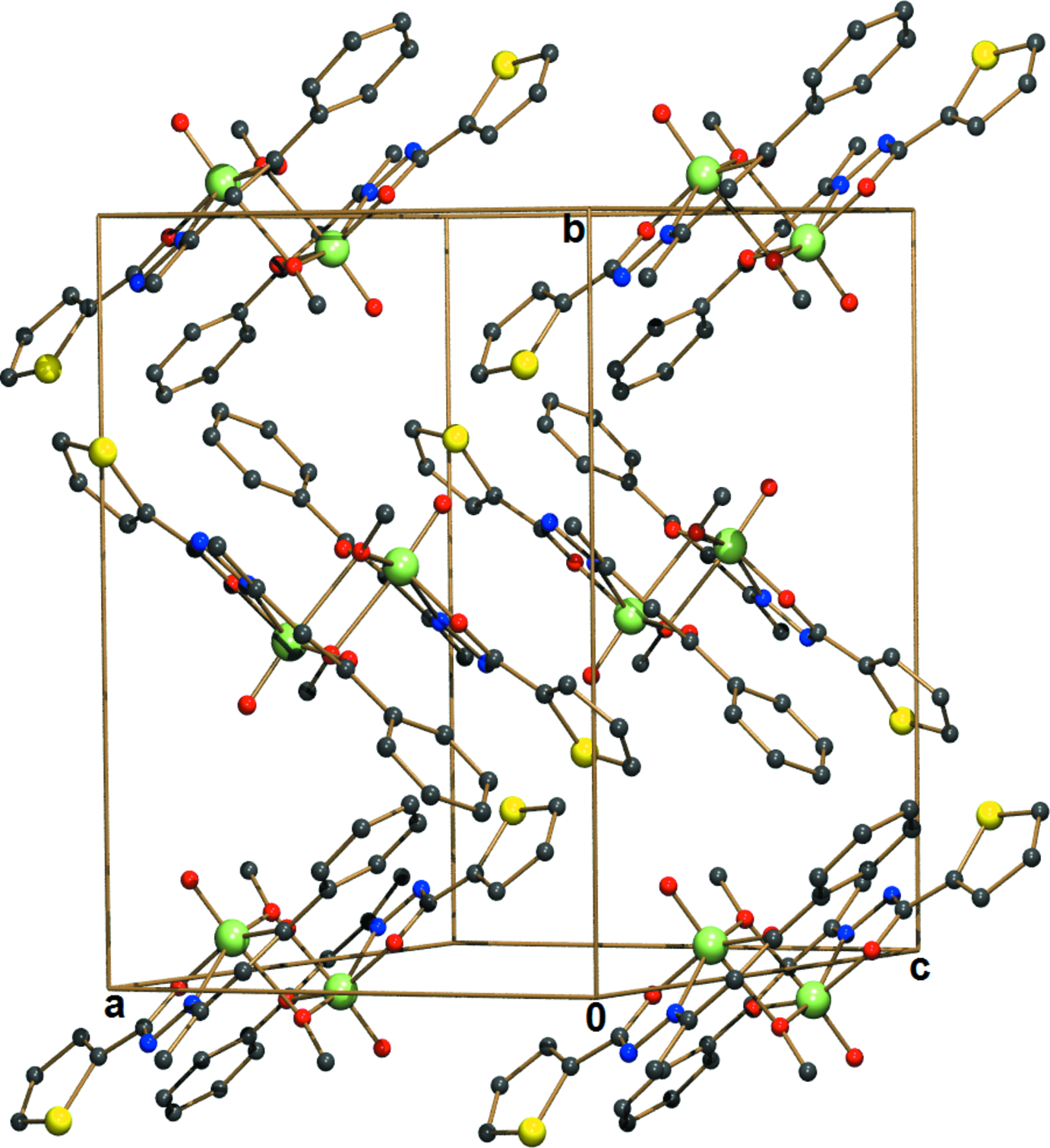

Supplement: Supplementary file 4 [file e-70-0m353-fig2.tif]
